# Validation of a Practical Approach to Blood Pressure Measurement: Secondary Analysis of Data from a Nationally Representative Survey in India

**DOI:** 10.5334/gh.1085

**Published:** 2021-12-22

**Authors:** Roopa Shivashankar, Bhawna Sharma, Andrew E. Moran, Anupam Khungar Pathni

**Affiliations:** 1 Resolve to Save Lives, Vital Strategies, Delhi, IN; 2 Resolve to Save Live, Vital Strategies, New York, US; 3 Columbia University Irving Medical Center, New York, US

**Keywords:** hypertension, screening, blood pressure measurement, primary health care, validation study

## Abstract

**Background:**

Clinical guidelines differ on the recommended number of blood pressure (BP) measurements for hypertension diagnosis in primary health care settings. We assessed the accuracy in identifying high BP (≥140/90 mmHg) and efficiency (mean BP measures per person in one visit) of a practical BP measurement approach against the research standard.

**Methods:**

We analyzed data from a national survey in India with three BP measurements for each adult participant (N = 372,110). The research standard (referred to as ‘standard approach’) is measuring three BP and using the mean of the last two. In the practical approach, the first BP reading was used if the measure was <140/90 mmHg; the second BP was used if the first BP was ≥140/90 mmHg. If the difference between either the first two systolic or diastolic BPs was >5 mmHg, then we used the third reading.

**Results:**

Prevalence of high BP was 15.5% and 14.9% using standard and practical approaches, respectively. The sensitivity, specificity, false positive, and false negative rates of the practical approach were 85.4%, 98.0%, 11.3%, and 2.7% compared to the standard approach. The practical approach was more resource-efficient (mean BPs/person/visit 1.4 versus 3.0 for the standard approach). The practical approach had similar validity, but higher efficiency compared to other internationally recommended BP measurement protocols.

**Conclusion:**

The practical BP measurement approach has high validity, is simpler and involves a lower measurement burden on health care providers and can improve the utility of BP measurement, hypertension diagnosis, and management in busy primary health care settings.

## 1. Introduction

Hypertension or high blood pressure (BP) is the number one risk factor for mortality globally and is responsible for more deaths than all infectious diseases combined [1]. Majority of the estimated 1.13 billion people with hypertension are in low- and middle-income countries (LMICs) wherein the primary health care systems deal with high patient volumes [2], often coupled with a limited number of trained health care workers [3]. It is estimated that there will be a shortage of 18 million health care workers worldwide by 2030, predominantly affecting LMICs [4]. With the increasing burden of non-communicable diseases in LMICs due to population growth and aging [2], hypertension care needs to be more efficient and patient-centered, otherwise the health systems will be overwhelmed by an increasing number of patients seeking care [5].

The quality of hypertension diagnosis and treatment decisions depends on the accurate measurement of BP. Single BP measurement is likely to over diagnose hypertension and therefore may lead to unnecessary medication [6]. The typical research standard is to take three BP readings and use the average of the last two readings as recommended by the International Society of Hypertension (ISH) 2020 and the European Society of Cardiology guidelines recommend the same for clinical practice [7,8]. Other international guidelines, including the American College of Cardiology/American Heart Association (AHA) 2017 and Eighth Joint National Committee (JNC-8) 2014, suggest simply averaging at least two BP measurements [6,9,10,11]. India’s National programme for prevention and control of cancer, diabetes, cardiovascular diseases, and stroke (NPCDCS) also recommends averaging a minimum of two BP readings [12]. Multiple BP measurements may not be practical in typical crowded primary health care settings in LMICs as the demands of multiple measurements in all patients and the need to calculate the average of the multiple BP readings not only requires more time from the health care providers but also potentially increases the risk of recording errors during averaging.

To address this, we suggest a practical approach for the number of BP measurements in one sitting at primary health care settings (**Box 1**). The rationale for the practical approach is based on the observations that (i) when multiple BP readings are taken, in 95% of the cases, the first measured BP reading is the highest [2,13] and (ii) if the first BP is higher than 140 mmHg systolic or 90 mmHg diastolic, using the second or third BP without averaging will result in a slightly lower recorded BP compared to the mean of all BPs. However, second or subsequent BPs are likely to be closer to the patient’s true mean BP (based on multiple BP measures; ‘regression to the mean’ phenomenon) [14].

**Box 1: Practical approach to blood pressure (BP) measurement (in mmHg)**

- If the first BP is <140 systolic AND <90 diastolic, then no other BP measurement is needed during that encounter. Use the first (and only) BP as the recorded BP.
- If the first BP is ≥140 systolic OR ≥90 diastolic, perform a second BP and use the second reading as the recorded BP for the encounter.
- If there is a large difference between the first and second systolic or diastolic reading (>5), use the third BP as the recorded BP.

The practical approach is similar to the existing recommendations by National Institute for Health and Care Excellence (NICE), United Kingdom [15], and may be sufficiently accurate to recommend for routine clinical practice. However, there are significant variations in terms of the design of a practical BP measurement algorithm. The NICE suggests the use of the lower of the 2^nd^ or 3^rd^ BP readings when 3^rd^ reading is taken while we recommend using only the 3^rd^ reading. A group at Johns Hopkins University recommends second reading if the first BP is ≥130/80 mmHg [16], while we recommend the 2^nd^ reading if the first BP is ≥140/90 mmHg. Any of these practical measurement approaches reduce the average number of readings per patient and reduce the cognitive burden to the health care provider.

In this paper, we assessed the performance of the ‘practical approach’ to BP measurement in comparison to the research standard (henceforth referred to as the ‘standard approach’). We assessed performance in terms of accuracy of the practical approach in identifying high BP and its grades (mild, moderate and severe) and efficiency in terms of the number of BP measurements required per patient per visit. Last, we assessed the accuracy and efficiency of three other commonly recommended approaches from the United States, United Kingdom, and India against the standard approach.

## 2. Methods

### 2.1. Data

We used data from a nationally representative household research survey [National Family Health Survey (NFHS)-4] conducted during 2015–16 in 640 districts of India [17]. The NFHS-4 used two-stage cluster random sampling stratified by urban and rural to select households. Primary sampling units -villages (rural) or census enumeration blocks (urban) were selected by population proportion to size. NFHS-4 included women in the age group of 15–49 years and men in the age group of 15–54 years. The BP was measured three times (after five minutes rest and a five-minute gap between the three measures) of the left arm (right arm if left arm is injured) in a sitting position using an automated BP measurement device (Omron HEM 8712) [18].

The following hypertension screening approaches were studied:

- Standard Approach: Measure three BP and use the average of the last two BP.
- Practical Approach: As described in Box-1.
- AHA/JNC-8 and NPCDCS approach: Average of first two BP measures [9,11].
- Johns Hopkins University approach: Use the first and only BP if the first BP < 130/80 and the second BP if the first BP ≥ 130/80 mmHg [16].
- The NICE approach: Use the first and only BP if the first BP < 140/90, use the second BP if the first BP is ≥ 140/90 mmHg; measure the third if the difference between first and second BP is large and use the lower reading of the second or third BP. Since the ‘large difference’ between in the second and third BP was not defined by NICE guidelines, we used the lower of the two if the difference was >5 mmHg for either systolic or diastolic BP [15].

### 2.2. Statistical Analysis

We excluded the observations if the age of the participant was <30 years as they have a lower risk of hypertension and most national programs (including India) recommend screening for ≥30 years [12]. We also excluded the observations with missing BP values in any of the three measurements or had unrealistic BP readings (systolic BP < 60 mmHg or >250 mmHg or diastolic BP < 30 mmHg or >250 mmHg or diastolic BP > systolic BP).

Regardless of the measurement approach, high BP was defined if the systolic BP ≥140 or diastolic BP ≥ 90 mmHg. We further stratified high BP as mild (BP 140–159/90–99), moderate (BP 160–179 or 100–109), and severe if BP (≥180/110). Age was stratified into two groups 30–44 and 45–54 years. The participants who reported to have received a diagnosis of hypertension were stratified as ‘known hypertension.’

We estimated the prevalence of high BP for the standard, practical, and other approaches as per the definition. We estimated sensitivity, specificity, and the misclassification of the high BP (false positivity and false negativity rates) categories by practical approach compared to the standard approach for all, by gender, and by age groups. Based on the highest sensitivity and specificity of both approaches, we estimated the positive predictive values (PPV) of correctly identifying high BP at different assumptions of the prevalence of high BP (0.10, 0.15, 0.20, 0.25, 0.30, and 0.35). The PPV was estimated using the formula: PPV = (sensitivity × prevalence)/[(sensitivity × prevalence) + ((1 – specificity) × (1 – prevalence))] [19].

We cross-tabulated the percentages of normal (BP <140/90 mmHg), mild (BP = 140–159/90–99), moderate (BP = 160–179/100–109), and severe (BP ≥ 180/110) high BPs of practical and standard approaches. We estimated the agreement between the two methods using the kappa statistic.

We estimated the average number of BP measurements needed per person. We first estimated the proportion of the participants needing 1, 2, and 3 BP readings and weighted each proportion to the number of the BP measurement and added them. For example, if 50% of the participants need only one BP, 25% need two, and another 25% need three BP, then the average BP reading person is 0.5*1 + 0.25*2 + 0.25*3 = 1.75. We estimated the average number of BP measurements for the total and sub-sample of those aged 45–54 years (largest prevalence).

We also compared the sensitivity, specificity, false positivity rate, and false negativity of AHA/JNC-8/NPCDCS, Johns Hopkins University’s and NICE approaches against the standard approach for identifying high BP.

We used sampling weights and estimated confidence intervals (CIs) adjusted for the primary sampling unit to get representative estimates.

## 3. Results

The NFHS-4 had collected data from 803,211 participants. We included the data of 372,110 participants after excluding 44,593 because of missing or unrealistic BP readings and 386,508 who were younger than 30 years of age. Of the 372,110 included, 55,896 (15%) were men, 285,415 (76.7%) were 30–44 years, 86,695 (23.3%) were in 45–54 years. A total of 44,508 (11.8%) had previously diagnosed hypertension and 17,235 (4.6%) reported taking medication for high BP.

Table 1 compares the prevalence of high BP and validity in categorising high BP by practical approach against the standard approach in all participants, and by gender and by age categories. The prevalence of high BP was 14.9% (14.7%–15.1%) and 15.5% (95% CI 15.2%–15.7%) using practical and standard approaches, respectively. The sensitivity and specificity of the practical approach compared to the standard approach to identify high BP was 85.4% and 98.0%, respectively. The sensitivity varied between 83.7% to 88.6% and specificity varied between 97.4% to 98.2% in different gender and age categories. The false positivity and false negativity rates varied between 9.1%–11.6% and 2.5%–3.4%, respectively. Based on the highest sensitivity of 88.6% and specificity of 98.2%, we estimated the positive predictive values for the different prevalence of high BP. The positive predictive values were 83.2%, 88.7%, 91.8%, 93.7%, 95.0% and 96.0% for high BP prevalence of 10%, 15%, 20%, 25%, 30% and 35%, respectively.

**Table 1:** Prevalence of high blood pressure (BP) and validity of practical BP measurement approach in identifying high BP (≥140/90) compared to standard approach.

	Sample size	Prevalence of high BP in % (95% Confidence interval)		Practical approach compared to standard (Percentages [95% CI])			

		Standard Approach	Practical approach	Sensitivity	Specificity	False positivity	False negativity

**All**	**372,110**	15.5 (15.2, 15.7)	14.9 (14.7, 15.1)	85.4 (84.9, 85.8)	98.0 (97.9, 98.1)	11.3 (10.9, 11.7)	2.7 (2.6, 2.7)
**Men**	55,896	21.5 (20.9, 22.1)	20.8 (20.3, 21.4)	87.3 (86.3, 88.2)	97.4 (97.1, 97.6)	10.0 (9.2, 10.8)	3.4 (3.2, 3.7)
**Women**	316,214	14.4 (14.2, 14.6)	13.8 (13.6, 14.0)	84.9 (84.4, 85.4)	98.1 (98.0, 98.2)	11.6 (11.2, 12.1)	2.5 (2.4, 2.6)
**30–44 years**	285,415	13.2 (13.0, 13.5)	12.7 (12.5, 12.9)	83.7 (83.1, 84.3)	98.2 (98.1, 98.3)	12.5 (12.0, 12.9)	2.5 (2.4, 2.6)
**45–54 years**	86,695	22.6 (22.2, 23.1)	22.0 (21.6, 22.5)	88.6 (87.9, 89.2)	97.4 (97.2, 97.6)	9.1 (8.5, 9.8)	3.3 (3.1, 3.5)

Table 2 provides the cross-tabulation of grading of high BP by the practical and standard approaches. The agreement between standard and practical approaches in stratifying the grades of hypertension was 94.7% (kappa = 0.807 [95% CI 0.806 and 0.811]; p-value < 0.0001).

**Table 2:** Crosstabulation of different grades of hypertension stratified by Practical and Standard blood pressure measurement approaches (N = 372,110).

Practical approach	N	Standard Approach (percentage [95% CI])			

		BP < 140/90	140–159/90–99	160–179/100–109	>180/110

**BP < 140/90**	314,221	97.3 (97.2, 97.4)	2.5 (2.5, 2.6)	0.1 (0.1, 0.1)	0 (0, 0)
**140–159/90–99**	42,141	15.3 (14.8, 15.8)	81.7 (81.2, 82.3)	2.9 (2.7, 3.2)	0 (0, 0.1)
**160–179/100–109**	11,324	0.1 (0.1, 0.2)	20.1 (19.0, 21.3)	76.3 (75.1, 77.5)	3.4 (2.8, 4.0)
≥**180/110**	4,424	0 (0, 0.1)	0.7 (0.4, 1.2)	15.9 (14.4, 17.5)	83.4 (81.8, 85.0)

The average number of BP measurements per person needed for the practical approach at high BP prevalence of 15% (from all data) and 23% (from sub-sample of ages 45–54 years) was 1.4 and 1.53, respectively (not shown in the table).

The prevalence of high BP by AHA/JNC-8/NPCDCS, Johns Hopkins University, and NICE approaches were 19.3%, 18.1%, and 14.6%, respectively. The average number of BP measurements per person using AHA/JNC-8/NPCDCS, Johns Hopkins University, and NICE approaches were 2.0, 1.6, and 1.4, respectively. The sensitivity, specificity, false positivity, and false negativity of AHA/JNC-8/NPCDCS, Johns Hopkins, and NICE approaches compared to the standard are provided in Table 3.

**Table 3:** Comparison of prevalence and validity of other various blood pressure (BP) measurement approaches against the Standard approach.

	High BP prevalence	Sensitivity	Specificity	False positivity rate	False negativity rate	No. of BP per person*

	Percentages [95% Confidence interval]					

**Standard**	15.5 (15.2, 15.7)					3.0
**Practical**	14.9 (14.7, 15.1)	85.4 (84.9, 85.8)	98.0 (97.9, 98.1)	11.3 (10.9, 11.7)	2.7 (2.6, 2.7)	1.4
**AHA/JNC-8/NPCDCS**	19.2 (19.0, 19.5)	93.4 (93.1, 93.7)	94.3 (94.2, 94.4)	25.1 (24.6, 25.6)	1.3 (1.2, 1.3)	2.0
**Johns Hopkins University**	18.1 (17.8, 18.3)	94.1 (93.8, 94.4)	95.9 (95.8, 96.0)	19.5 (19.0, 20.0)	1.1 (1.1, 1.2)	1.6
**NICE**	14.6 (14.3, 14.8)	84.5 (84.0, 85.0)	98.2 (98.1, 98.3)	10.5 (10.1, 10.9)	2.8 (2.7, 2.9)	1.4

* Average number of BP measurements per person per visit.

## 4. Discussion

In LMICs, in addition to the need for accurate measurement of BP, given the large volume of patients and high patient-to-health care provider ratio at primary care facilities, improving the efficiency of BP measurement is a dire need. Further, the approach to hypertension screening should minimize cognitive burden, [20] specifically when more and more countries and programs are task-shifting hypertension care to non-physician health workers [21]. While the provision of validated BP instruments and training of health care staff on the correct BP measurement technique addresses the measurement accuracy, there is also a pressing need to simplify the BP measurement approach to improve efficiency.

We tested a practical approach to BP measurement and found it resulted in lower mean BP measurements per patient whilst preserving accuracy. The practical approach had moderate-high sensitivity and high specificity in identifying high BP when compared to the standard approach. Despite the fact that this approach requires only a single BP measurement in most participants, it had a low false positivity rate against the standard as compared to other approaches (except for NICE, which also used single BP measurement for most participants). This is reassuring because researchers have raised concerns about the overestimation of hypertension when using a single BP measurement in a recent paper from India using the same NFHS dataset [6]. Further, the practical approach requires less than two BP measurements per person even in a scenario with a high prevalence of hypertension, suggesting that it may perform equally well in other populations. Hence it is likely to improve compliance to BP measurement guidelines among healthcare workers.

The practical approach was developed to ensure the least cognitive burden on the health care provider measuring BP in the busy clinics. There are two main cognitive burdens in the different approaches recommended for BP measurement. One, averaging two or more BP readings, which requires mathematical calculation (as in standard approach and AHA/JNC-8/NPCDCS approaches). In addition to increasing the time burden, averaging can lead to errors in the calculation in busy clinics. Further, the average of the first two BPs had high false positivity rate and had similar concerns relating to overdiagnosis as of a single BP measurement [6]. Two, identifying the lowest of the two or more BP measures (as in the NICE approach). This can be confusing if one of two measures, that is systolic or diastolic BP is high and other is low. Three, having different BP cut-offs for determining when to take the second measurement. The Johns Hopkins’ approach suggests taking a second BP reading if the first reading is greater than 130/80 mmHg. This would require health care workers to memorise two different threshold values for taking the second measurement and suspecting or diagnosing high BP.

Our study has several strengths. One, we used a large dataset with valid BP measurements from a LMIC. Two, we compared the practical approach with the most common other globally recommended approaches. Three, we assessed how the different approaches performed in categorising the BP as high. While the parametric measure of mean differences in BP by different approaches is statistically superior, the clinical decisions for diagnosis and titration of medication relies on BP categories. Our study did have limitations. One, we used data from a single country, India. Nevertheless, the sample size of the study was huge, and the findings of the study have high external validity. Two, the data is from a population survey where participants are likely to be low-risk and have lower BP values (as there is no white-coat hypertension) compared to a typical clinic population. As expected, the positive predictive value of the practical approach increased with the increasing prevalence of high BP. Therefore, the practical approach is likely to perform better in clinics than what is shown in this analysis. Finally, the regression to mean phenomenon in multiple measures of BP had found that subsequent BPs may also be higher than the first BP, therefore the practical approach may miss some persons with high BP [22,23]. This phenomenon manifested in the slightly higher false negative rate in the practical approach compared to other approaches. However, these estimates are cross-sectional. Most adults will be re-screened over time, which would increase the proportion of true positive diagnoses in a cohort. Overall, we contend that the improved efficiency in BP measurement using the practical approach will over time increase the detection and management of hypertension and compensate for the smaller false negative rate.

## 5. Conclusion

A simplified, practical approach to BP measurement has high validity and acceptable agreement with the standard approach. The practical approach has the potential to improve the efficiency of BP measurement in primary health care settings, increasing primary health care facility capacity to diagnose and manage raised BP in a large number of hypertension patients indicated for treatment. This approach also provides the advantages of lower work time and cognitive burden on health care workers. The validity and feasibility of the approach should be tested in pragmatic settings through implementation research methodology.

## Data Accessibility Statement

NFHS data used for this study is downloadable at: https://www.dhsprogram.com/.
